# The links between diabetes mellitus and amyotrophic lateral sclerosis

**DOI:** 10.1007/s10072-021-05099-0

**Published:** 2021-02-05

**Authors:** Rosario Vasta, Fabrizio D’Ovidio, Giancarlo Logroscino, Adriano Chiò

**Affiliations:** 1 ALS Center, ‘Rita Levi Montalcini’ Department of Neuroscience, University of Turin, Turin, Italy; 2 Department of Clinical Research in Neurology, Center for Neurodegenerative Diseases and the Aging Brain, University of Bari “Aldo Moro”, “Pia Fondazione Cardinale G. Panico”, Tricase, Italy; 3 Department of Basic Medical Sciences, Neuroscience and Sense Organs, University of Bari “Aldo Moro”, Bari, Italy; 4 Neurology 1, Azienda Ospedaliero Universitaria Città della Salute e della Scienza, Turin, Italy

**Keywords:** Amyotrophic lateral sclerosis, Epidemiology, Diabetes mellitus, Metabolic syndrome

## Abstract

ALS etiology and prognostic factors are mostly unknown. Metabolic diseases and especially diabetes mellitus (DM) have been variously related to ALS. However, pieces of evidence have been variegated and often conflicting so far. This review aims to give an overview of recent contributions focusing on the relationship between DM and ALS. DM seems to reduce the risk of developing ALS if diagnosed at a younger age; conversely, when diagnosed at an older age, DM seems protective against ALS. Such a relationship was not confirmed in Asian countries where DM increases the risk of ALS independently of the age of onset. Interestingly, DM does not affect ALS prognosis, possibly weakening the potential causal relationship between the two diseases. However, since most studies are observational, it is difficult to state the exact nature of such a relationship and several hypotheses have been made. A recent study using Mendelian randomization suggested that DM is indeed protective against ALS in the European population. However, these analyses are not without limits and further evidence is needed. DM is usually the core of a larger metabolic syndrome. Thus, other metabolic changes such as dyslipidemia, body mass index, and cardiovascular diseases should be collectively considered. Finally, hypermetabolism usually found in ALS patients should be considered too since all these metabolic changes could be compensation (or the cause) of the higher energy expenditure.

## Introduction

Amyotrophic lateral sclerosis (ALS) is a neurodegenerative disease affecting motor neurons. It causes the paralysis of voluntary muscles, leading to death, usually for respiratory failure, in 2 to 4 years from symptoms onset [1]. In most cases, the etiology of ALS is unknown but it is thought to involve both genetic and environmental factors [1]. The difficulty in identifying risk factors for ALS reflects the complexity of the disease, and a more systemic approach may be needed to unravel significant associations with genetic and environmental factors [2].

Diabetes mellitus (DM) is a chronic disease caused by dysfunctional use, or the lack, of insulin and resulting in impaired blood glucose regulation. DM can be classified as type 1 (T1DM) and type 2 (T2DM) depending on the deficient insulin production or on the body’s ineffective use of insulin, respectively [3]. DM has an 8.5% prevalence among the adult population; in 2014, 422 million people worldwide were affected by DM [4].

Given the hypermetabolism that accompanies ALS [5], the association with concomitant metabolic diseases such as DM, hypertension, dyslipidemia, and obesity has been diffusely studied [6]. In this review, we aimed to examine all contributions focused on the association between DM and ALS.

## Methods

We performed a review of peer-reviewed articles in full and only in English. The searching process was performed in PubMed MEDLINE, using the following search formula: (“ALS” OR “Amyotrophic Lateral Sclerosis” OR “motor neuron disease” OR “Lou Gehrig disease”) AND (“diabetes” OR “Diabetes mellitus” OR “Glucose intolerance” OR “insuline” OR “hyperglycemia”). The review design was not restricted by study design. The eligible criteria were firstly evaluated by abstract readings and subsequently by reading the remaining articles. References of the collected published studies were also considered. Because of the high number of articles retrieved, searching was restricted to a 7-year period, starting from January 1, 2013, and updated to December 31, 2019. Given the diversity of study designs and measures of associations, a narrative review was performed.

## Results

A total of 230 articles were identified. Of these, 35 articles were considered pertinent for the narrative analysis.

The more frequent article types were cohort studies (*n* = 10) followed by case-control studies (*n* = 7), reviews (*n* = 5), and letters (*n* = 4). Editorials (*n* = 3), cellular (*n* = 2), and animal studies (*n* = 2) were also retrieved, together with 1 observational cohort study protocol and 1 randomized clinical trial (Table 1).

**Table 1:** Articles included in the review

Reference	First author, year	Type of study	Nation	Diabetes as risk/prognostic factor	Effect size
[6]	Brito, 2019	Review	-	Prognosis	-
[7]	Mitchell, 2015	Case-control	USA	Risk	OR 0.47 (95%CI (0.38-0.58))
[8]	Mariosa, 2015	Nested case-control	Sweden	Risk	Overall: OR 0.79 (0.68–0.91) < 50 age class: OR 3.15 (1.40–7.08) 70–79 age class: OR 0.71, 95% CI 0.57–0.89 ≥ 80 age class: OR 0.56, 95% CI 0.40–0.78
[9]	Kawada, 2016	Letter to the Editor	-	-	-
[10]	Mariosa, 2016	Letter to the Editor	-	-	-
[11]	Kioumourtzoglou, 2015	Nested case-control	Denmark	Risk	Overall: OR 0.61 (95% CI 0.46-0.80) At 65 years old: OR 0.65 (95% CI 0.50–0.85) At 35 years old: OR 1.68 (95% CI 0.75–3.75)
[12]	Sun, 2015	Cohort	Taiwan	Risk	Overall: HR 1.35 (95% CI 1.10–1.67) Men < 65 years old: HR 1.67; 95% CI 1.18–2.36
[13]	Tsai, 2019	Cohort	Taiwan	Risk	Overall: HR 0.87, 95% CI 0.70–1.07 ≥ 55 years age class: HR 0.72, 95% CI 0.55–0.95
[14]	Seelen, 2014	Case-control	Netherlands	Risk	OR 0.72, 95% CI 0.51–1.01
[15]	D’Ovidio, 2018	Cohort	Italy	Risk	HR 0.30, 95% CI 0.19–0.45
[16]	Logroscino, 2015	Editorial	-	-	-
[17]	Turner, 2013	Cohort	England	Risk	RR 3.94; 95% CI 1.84–7.50
[18]	Jawaid, 2015	Editorial	-	-	-
[19]	Korner, 2013	Cohort	Germany	Prognosis	On survival: HR 1.11, 95% CI 0.76–1.60 On progression: HR 1.07, 95% CI 0.74–1.57
[20]	Paganoni, 2015	Cohort	RCTs	Prognosis	Not provided
[21]	Jawaid, 2015	Letter to the Editor	-	-	-
[22]	Paganoni, 2015	Letter to the Editor	-	-	-
[23]	Mandrioli, 2018	Cohort	Italy	Prognosis	HR 1.11, 95% CI 0.93–1.33
[24]	Moglia, 2017	Cohort	Italy	Prognosis	HR 1.05, 95% CI 0.78–1.42
[25]	Wei, 2017	Cohort	China	Prognosis	HbA1c between 5.7% and 6.4%: HR 1.40 HbA1c > 6.5%: HR 2.06
[26]	Zhang, 2019	Cohort	China	Prognosis	HR 0.84, 95% CI 0.68–1.30
[27]	Hollinger, 2016	Case-control		Prognosis	Not provided
[28]	Zeng, 2019	Case-control	Europe/Asia	Risk	Europe: OR 0.93, 95% CI 0.88–0.99 Asia: OR 1.17, 95% CI 0.93–1.47
[29]	Lim, 2014	Animal study	-	-	-
[30]	Joardar, 2017	Review	-	-	-
[31]	Rauskolb, 2017	Review	-	-	-
[32]	Shi, 2019	Animal study	-	-	-
[33]	Araki, 2019	Cellular study	-	-	-
[34]	Jawaid, 2018	Editorial	-	-	-
[35]	Pfeiffer, 2019	Case-control	USA	-	-
[36]	Walker, 2016	Protocol	UK	-	-
[37]	Liu, 2015	Review	-	-	-
[38]	Liu, 2015	Cellular study	-	-	-
[39]	Wills, 2014	RCT	-	-	-
[40]	Herskovits, 2013	Review	-	-	-

Studies not reporting an explicit link between DM and ALS are not included in this table. *OR*, odds ratio; *RR*, relative risk; *HR*, hazard ratio; *95% CI*, 95% confidence intervals; *RCT*, randomized clinical trial; HbA1c, glycated hemoglobin

Studies not reporting an explicit link between DM and ALS were not included in this count.

However, other contributes were also examined in order to better understand the mechanisms behind the association between DM and ALS.

Because of the high diversity of these results, the review was structured into thematic sections.

### Diabetes mellitus as a risk factor for ALS

In the USA, a case-control study enrolling 1288 cases and 7561 controls reported a protective role of DM as antecedent condition of ALS (odds ratio (OR) 0.47; 95% confidence intervals (CI) 0.38–0.58) [7].

In a Swedish nested case-control study which enrolled 224 ALS cases and 1437 controls individually matched for age, sex, and area of residence, DM was found to be protective against ALS but only among subjects above 70 years (OR 0.71, 95% CI 0.57–0.89 in the 70–79 age class and OR 0.56, 95% CI 0.40–0.78 in the > 80 age class), whereas below 50 years DM was associated with an increased ALS risk (OR 3.15, 95% CI 1.40–7.08) [8]. However, two editors’ letters pointed out the lack of adjustment of body mass index (BMI) in the analyses, the exclusion of patients with T1DM, and the role of drug medication on the association between T2DM and ALS [9, 10].

Similar results were obtained by a Danish nested case-control study, which enrolled 3650 ALS patients and 365,000 controls extracted from the Danish National Register system, matched for sex and age [11]. The study reported an overall inverse association between DM and ALS (OR 0.61, 95% CI 0.46–0.80), with a protective effect of DM above 61 years (at 65 years old OR 0.65, 95% CI 0.50–0.85) and a non-significant increased risk below 51 years (at 35 years old OR 1.68, 95% CI 0.75–3.75). Another cohort study was conducted in Asia, enrolling 615,492 diabetic subjects and 614,835 subjects as comparison cohort matched for age and sex followed up from 2000 to 2008 [12]. Authors found an overall significant hazard ratio (HR) of 1.35 (95% CI 1.10–1.67), with sex and age as modifiers of the association: DM was significantly and positively associated with ALS only for men younger than 65 years old (HR 1.67; 95% CI 1.18–2.36).

Finally, a recent retrospective cohort study was conducted in Taiwan using administrative data and considering patients who had a T2DM diagnosis during the 2000–2013 period (*n* = 2,135,427) compared to a matched sample from the unexposed population. The risk of developing ALS did not differ in patients with prior T2DM (HR 0.87, 95% CI 0.70–1.07, *p* = 0.190). However, when considering the age at T2DM diagnosis, DM resulted negatively associated with ALS in patients whose age was ≥ 55 years (HR 0.72, 95% CI 0.55–0.95, *p* = 0.019) [13].

In a large Dutch case-control study, DM inverse relationship with ALS resulted slightly not significant (OR 0.72, 95% CI 0.51–1.01) [14].

Such opposite risk based on age at T2DM diagnosis was not confirmed by a recent Italian cohort study, which followed 727,977 residents in Turin from 1996 to 2014 and included a time-dependent analysis. DM resulted to be significantly associated with a decreased risk of ALS (HR 0.30, 95% CI 0.19–0.45), without significant differences in risk by sex, age, and ALS phenotype [15].

The inverse relationship between DM and ALS depending on age at DM diagnosis was underlined in a recent editorial [16]. The author also argued that mixed ethnic groups showed a lower ALS risk than people of African or European ancestry even when they live within the same geographic area, thus suggesting a possible role of genetics in such differences [16].

All these studies focused on T2DM. A British study assessing the risk of ALS for people affected by each of a range of autoimmune diseases showed that T1DM did not confer an increased risk of developing ALS. However, ALS risk resulted significantly increased for people aged less than 30 years at first diagnosis of T1DM (relative risk (RR) 3.94; 95% CI 1.84–7.50) [17]. A previous study distinguished T1DM and T2DM only based on the age at diagnosis, respectively under and above 30 years age [8]. Both types resulted to be inversely associated with ALS, but only T2DM was significantly associated (OR 0.65, 95% CI 0.52–0.79).

An editorial based on this study highlighted the opposite effect of T1DM and T2DM in the developing ALS, and the role of BMI correlated with both types of DM, and also with ALS onset and survival [18].

### Diabetes mellitus as a prognostic factor for ALS

A German study reconstructed comorbidities of a cohort of 514 ALS patients in order to estimate their effect on survival and disease progression, revealing that DM did not significantly affect both survival (considered as the interval from ALS diagnosis to death, tracheostomy, or censoring, HR 1.11, 95% CI 0.76–1.60) and progression (estimated by using the logarithmic ALSFRS-R score ratio as the dependent variable, HR 1.07, 95% CI 0.74–1.57) [19]. A study including 1322 patients from 6 ALS clinical trials showed that survival was not different from DM to non-DM patients (*p* = 0.98) [20]. Regarding this study, a letter to the editor [21] and a reply [22] were written in order to better clarify the methodological differences between this and another American study [41].

Even in two recent Italian studies, DM was not significantly associated with ALS survival. The first one was performed in 13 Italian ALS centers from 2009 to 2013 and enrolled 2354 incident ALS patients. The authors reported a non-significant effect of DM on ALS survival (HR 1.11, 95% CI 0.93–1.33) [23]. In the second population-based cohort study, 650 ALS incident patients were recruited and T2DM was not associated with survival (HR 1.05, 95% CI 0.78–1.42; *p* = 0.84) [24].

A Chinese cohort study recruiting 450 ALS patients indicated that higher levels of hemoglobin A1c (HbA1c) at diagnosis were significantly associated with a higher risk of mortality (HR 1.40 for HbA1c between 5.7 and 6.4% and HR 2.06 for HbA1c > 6.5%; *p* trend = 0.01) [25]. Finally, in another study, survival was not significantly longer in the diabetic ALS patients group when compared to non-DM patients (HR 0.84, 95% CI 0.68–1.30; *p* =0.617) [26].

Notably, a recent systematic review pointed out that pieces of evidence are not sufficient to establish a link between metabolic alterations and ALS progression [6].

DM was also found to delay ALS onset. A case-control study focusing on antecedent conditions included 1439 ALS patients distinguished in 600 patients without antecedent conditions and 839 patients with at least one antecedent condition. DM was found to be significantly associated with both later ages of ALS onset (*p* < 0.0001) and shorter disease duration (*p* < 0.0069) [27]. Same results were reported also in a previous study, which evaluated 2371 ALS patients and where DM was associated with a 4-year later onset of ALS (56.3 years old for non-DM ALS patients and 60.3 years old for diabetic ALS patients, *p* < 0.05) [41]. Such delay was confirmed by a Chinese study including a total of 2562 ALS which found, after adjusting for sex and site of onset, that patients with pre-morbid DM showed a 4.4-year delay in the ALS onset (57.0 ± 9.6 vs 52.6 ± 10.3 years; *p* < 0.001) [26].

### Genetic links

In DM, hereditary components are estimated to range between 20 and 80% [42]. The first genes revealed were *CAPN10* and *TCF7L2*, but other genes were considered candidates to play a significant role in the pathogenesis of T2DM, such as *PPARG*, *IRS1*, and *IRS-2*, *KCNJ11*, *WFS1*, *HNF1A*, *HNF1B*, and *HNF4A* [42].

Over the past 20 years, several gene mutations in ALS have been identified, including both major genes (such as *SOD1*, *TARDBP*, *FUS*, *OPTN*, *VCP*, *UBQLN2*, *C9ORF72*, and *PFN1*) and several other minor genes [43]. Among minor genes, *SH2B3* and *ATXN2* were found to be associated with both autoimmune and neurodegenerative diseases [44]. *ATXN2* plays an important role for ALS since intermediate-length polyQ expansions (27–33 glutamines) in this gene were found to be significantly associated with the ALS risk [45]. Furthermore, a Turkish study, in which genomes of 158 sporadic and 78 familial ALS patients were compared with those of 420 healthy controls, found that 31–32 polyQ repeats in the *ATXN2* gene were associated with a risk of ALS in 1.7% of the Turkish ALS cohort (*p* = 0.0172) and that a significant association of a 136-kb haplotype block across *ATXN2* and *SH2B3* genes was found in 19.4% of a subset of the ALS cohort and in 10.1% of the controls (*p* = 0.0057, OR 2.23), an indication that *ATXN2* and *SH2B3* variants may interact in modulating the disease pathway [46].

A recent study analyzed GWAS data from two large European and Asian samples (~ 660,000 individuals for T2DM and ~ 81,000 individuals for ALS in the European population, and ~ 191,000 individuals for T2DM and ~ 4100 individuals for ALS in the East Asian population). Adopting the Mendelian randomization (MR) approach, authors demonstrated that single-nucleotide polymorphisms associated with T2DM were negatively associated with ALS in the European sample, therefore suggesting that T2DM might be actually protective for ALS (OR 0.93, 95% CI 0.88–0.99, *p* = 0.023), rather than being the effect of potential confounders or reverse causality. An opposite despite no significant relationship was found in the Asian sample (OR 1.17, 95% CI 0.93–1.47, *p* = 0.190). However, the authors stated that the smaller size of the Asian sample could account for the lack of significance [28].

### Pathogenic links

The biological mechanisms linking DM to ALS remain unclear but it is likely that energy metabolism and homeostasis should be taken into account [5]. About 50% of ALS patients show hypermetabolism (i.e., an increased energy expenditure) compared with controls and this could justify a higher consumption of glucose and lipids [47].

In a preclinical study setting, the correction of defects in energetics through a high-fat diet in mutant *SOD1* mice delayed the ALS onset, improved the overall survival, and reduced muscle denervation [48]. A further study in mutant *SOD1* mice reported that ALS progression could be mitigated by altering energy metabolism [29]. In particular, the altered energy metabolism reduced circulating leptin levels, an adipocyte-derived hormone that regulates the whole-animal energy expenditure, which in turn decreased the rate of weight loss, increased the white adipose tissue stores, decreased motor neuron degeneration, and finally improved survival. Furthermore, people affected by T2DM reported higher concentrations of progranulin [49], an adipokine that mediates high fat-induced insulin resistance and whose overexpression has been shown to revert mutant TDP-43-induced axonopathy in vivo [50].

In this optic, higher serum lipids or glucose could compensate ALS patients’ hypermetabolism, thus reducing the rate of the damage caused by the hypermetabolic state on the motor neuron system [47] and delaying ALS onset [41].

*TBK1* is considered among ALS minor genes and codes for the TANK-binding kinase 1[30]. In a genetic mouse model, TBK-1 has been proved to contribute to the phosphorylation of the insulin receptor, eventually attenuating its functionality. This evidence suggests that TBK1 could be involved in in vivo insulin resistance [51].

A recent review suggested the need to link together basic research with personalized medicine approaches to define new therapies based on cellular energetics in ALS [52]. However, alternative hypotheses should be also considered.

Uric acid has been suggested to predict survival in ALS and has also been positively associated with DM in high concentrations; thus, it could be considered as a potential pathogenic link between DM and ALS [11].

Glutamate excitotoxicity has been linked to motor neurons’ death. Conversely, hyperglycemia has been suggested to increase glutamate uptake, thus protecting against excitotoxicity [11].

Vitamin A metabolism has been also studied. Indeed, high concentrations of serum retinol-binding protein 4 (RBP4), a specific transport protein of Vitamin A, resulted to be inversely correlated with the risk of ALS (OR 0.36, 95% CI 0.22–0.59). RBP4 has been investigated and has a proxy of insulin resistance [31]. Furthermore, retinoic acid, a metabolite of vitamin A playing an important role in the development and programmed cell death, was reported as relevant to the pathogenesis of ALS also in other studies [53].

A recent review focused on the role of insulin-like grow factor 1 (IGF-1) in both DM and ALS [54]. IGF-1 has a 50% sequence homology with insulin and is able to elicit nearly the same hypoglycemic effects [55]. Several studies have reported an increased risk of insulin resistance and T2DM in subjects with low IGF-1 serum concentrations [56]. IGF-1 also promotes the survival of neurons and supports axon growth and has been shown to be lower in the cerebrospinal fluid of ALS patients when compared to controls (*p* < 0.0001) [57].

However, it should be highlighted that three randomized clinical trials (RCTs) have been conducted to test the disease-modifying effect of the recombinant human IGF-1 (rhIGF1) on ALS [58–60]. All these trials showed no clear beneficial effect of IGF-1 on ALS progression, whereas a meta-analysis concluded that, considering the two RCTs using the same outcome measure [58, 60], a significant difference in favor of rhIGF-1 treatment was shown; however, the quality of the evidence from the two trials was low [61]. These results make it less probable for IGF-1 to play a crucial role in ALS pathogenesis and to represent the pathogenic link with DM.

An interesting review showed that chronically prolonged endoplasmic-reticulum (ER) stress is a hallmark of many common neurodegenerative and metabolic diseases, such as ALS and DM [62]. Several studies showed that ER stress occurs in motor neurons of human ALS patients [32]. Alteration in the ER functionality could lead to the production of unfolded proteins (UP) and UP response has been suggested to participate in carbohydrate metabolism [33].

A recent study focused on immune-mediated mechanisms. Altered humoral immunity was found to stimulate a pathological voltage-dependent Ca2+ entry in ALS motor neurons, thereby damaging these cells through a Ca2+ toxicity. Based on the evidence that motor neurons and islet cells share some mechanisms such as Ca2+-dependent exocytosis and triggered cell death, the study showed that IgG from ALS patients was able to interact with rodent islet cells, causing an Ca2+-mediated impairment of mitochondrial function, insulin secretion, and cell viability [34].

Interestingly, in a recent study, an impaired insulin secretion in the early phases of the disease and a nuclear depletion of TDP-43 in pancreatic beta-cells of ALS patients have been demonstrated. The loss of TDP-43 was prominent in beta-cells when compared to alpha-cells, thus suggesting a specific role of TDP-43 in insulin secretion. Furthermore, when knocking down the *TARDBP* gene in a cultured beta-cells line, insulin secretion was inhibited, possibly through the downregulation of Ca2+ channels. Notably, ALS patients enrolled in this study had normal basal insulin secretion levels but lower insulinogenic index (IGI), an index of early-phase insulin secretion [35].

TDP-43-positive cytoplasmic inclusions can be found in almost all ALS cases [1]. TDP-43 has been shown to influence fat accumulation and insulin sensitivity in both the liver and the skeletal muscle [16] and these results further suggest its extra-neurological role.

### Implications for ALS treatment

A recent editorial focused on the need for treatments for ALS and on identifying a clear biological mechanism explanation for the association between DM and ALS [36]. However, it should be first clarified whether anti-diabetes drugs rather than DM itself could play a role in ALS risk and progression. An observational study showed that a wide group of diabetes drugs was associated with a decreased risk of developing ALS, thus suggesting their possible “repurposing” [63]. Several experimental pieces of evidence for the use of diabetes drugs in ALS have been collected so far.

#### Pioglitazone and metformin

A protocol of an observational cohort study was published in order to investigate whether prescribed drugs for the treatment of T2DM, among others, could be associated with the risk or the progression of several neurodegenerative diseases, including ALS [64].

Based on the decreased levels of inflammatory mediators in transgenic mouse models and the anti-oxidant and anti-inflammatory effects of pioglitazone, a phase II, double-blind, multicentre, placebo-controlled trial on this drug (45 mg/day) in 219 ALS patients treated with riluzole was conducted. However, pioglitazone did not confer a benefit on survival (HR 1.21, 95% CI 0.71–2.07) [65]. Similar findings resulted in another study on the transgenic ^*G93A*^*SOD1* mice exposed to metformin, another anti-diabetic drug with potent anti-inflammatory, and anti-oxidative proprieties, which showed a dose-dependent negative effect on the disease progression in female mice (*p* =  0.036) [37].

#### AMPK activators

The enzyme AMP-activated protein kinase (AMPK) is a master regulator of energy balance [66]. AMPK is a common target for anti-diabetic drugs (for example, metformin) and the abnormal activation of AMPK was found in some neurodegenerative diseases, such as Alzheimer’s disease (AD), Parkinson’s disease, Huntington’s disease (HD), and ALS, possibly because of its role in the autophagy network [67]. In the ^G93A^*SOD1* mice, the activation of AMPK via the use of latrepirdine resulted in a delayed symptom onset and a significant increase in lifespan (*p* < 0.01) [38], while an increased AMPK activity seems to play a negative role in motor neuron survival in another animal model [68].

In another preclinical study using TDP-43 transgenic mice, the downregulation of the α1 subunit of AMPK played a relevant role in reducing TDP-43 mislocalization and the development (and progression) of ALS, suggesting AMPK-α1 as a potential drug target [69]. The aberrant activation of AMPK can drastically impact the normal distribution of the human antigen R (HuR, a major mRNA stabilizer), which may imbalance RNA metabolism and contribute to ALS pathogenesis [70].

AMPK activators are widely prescribed to DM patients and should be further investigated as potential therapeutic strategies.

#### Dietary modifications

A Japanese case-control study revealed that combined high intakes of carbohydrate (adjusted OR 2.14, 95% CI 1.05–4.36; the highest versus the lowest tertile) and low intakes of total fat (adjusted OR 0.41, 95% CI 0.21–0.80; the highest tertile versus the lowest) may increase the risk of ALS, suggesting that high-fat diet could be instead protective [39]. According to this hypothesis, in a mouse model, the administration of a ketogenic diet led to an improvement of motor neuron survival and motor function [71]. Furthermore, a human study demonstrated that among patients treated with high caloric enteral diet, those who received high carbohydrate had a smaller total number of adverse events (0 versus 9) and death (0% versus 43%) than those received high fat or control group [40], in keeping with the hypothesized protective role of hyperlipidemia on ALS survival [27].

#### The sirtuins pathway

Mammalian sirtuins are a group of seven NAD+-dependent enzymes able to deacetylate many intracellular proteins involved in many processes, including carbohydrate and lipid metabolism, apoptosis, and autophagy [72]. Accordingly, sirtuins were suggested to be protective against AD, HD, and ALS, via several mechanisms like regulation of stress response, apoptosis, and DNA repair [71, 73]. Moreover, SIRT2 was found to be inversely associated with mitochondrial fragmentation and neuronal cell death in ^G93A^*SOD1* transgenic mice [74]. Sirtuin activators have been proposed as a therapeutic target in DM and could be considered as a potentially valuable therapeutic factor in ALS [73].

## Discussion

Recent literature converged into stating that DM diagnosed at younger ages has a detrimental or null effect on the development of ALS; when DM is diagnosed at older ages (about 50–60 years), as it happens more commonly, it shows a protective effect on the ALS risk. There is no clear explanation for this age effect and some studies did not confirm it [15].

A possible explanation is that this age cutoff excludes most T1DM cases that rely on different pathogenic mechanisms [8]. However, such a phenomenon should be further studied since the distinction between T1DM and T2DM solely on basis of age could be imprecise [3].

Also, competing DM and ALS risks at older ages should be taken into account as a possible explanation.

The protective role of DM on ALS was not confirmed in the East Asian population where T2DM seems to increase the risk of ALS [12, 28]. It is plausible to think that genetic and environmental factors could act differently across populations, thus varying the terms of this interaction [16]. However, only two studies provided such opposite results and further pieces of evidence should be sought.

It remains unclear whether T2DM and ALS are linked by a causal relationship. Indeed, because of the retrospective observational nature of all these studies, despite most of them have attempted to adjust for some known confounding factors, it is impossible to completely rule out known not considered and unknown potential confounders. Conversely, the low frequency of ALS and obvious ethical aspects hamper observational prospective and experimental studies. Mendelian randomization (MR) could give useful hints in the attempt of moving beyond these limits and the study by Zeng et al. could be considered as proof of the causal relationship of diabetes on ALS [28]. However, MR is not without intrinsic limitations and it cannot entirely rule out the role of possible confounders and reverse causation [28, 75]. Therefore, it remains still unclear if T2DM could be considered as protective to ALS, if ALS causes the lack of T2DM (together with other metabolic conditions) or if both are caused, with opposite relationships, by a third not considered confounding factor.

In the light of a possible repurposing of drugs acting on glucose metabolism, clarifying the relationship between DM and ALS becomes crucial. Some preclinical studies showed positive results so far [31, 38, 70]. However, in a sensitivity analysis, Zeng et al. showed that fasting glucose, fasting insulin, and Hb1Ac do not share a causal relationship with ALS [28]. Thus, intervening on such factors could be ineffective and other targets, maybe occurring upstream in the pathogenic pathways, should be investigated. Together with the heterogeneity of ALS, this observation could justify the negative results of some trials using anti-diabetic drugs for modifying the risk of ALS. Furthermore, the possible opposite effect of DM on ALS in Europe and Asia could impose different therapeutic strategies in these two populations.

Interestingly, despite suggested as a protective factor, several studies showed that T2DM does not affect ALS prognosis. Such discrepancy could be interpreted as a lack of causal relationship between DM and ALS. However, it is not possible to exclude that ALS pathogenesis involves several pathways at different times, each one showing variable vulnerability to DM.

DM is usually part of a wider modification of metabolism known as metabolic syndrome [76]. Thus, focusing on the relationship of ALS with DM could mean missing the big picture. The most widely accepted hypothesis considers insulin resistance as the pathogenic center of metabolic syndrome [76]. Insulin inhibits lipase thus allowing the delivery of free fatty acids (FFAs) from the adipose tissue. In turn, FFAs inhibit, by a negative feedback mechanism, the action of insulin. This justifies how insulin resistance could lead to the accumulation of adipose tissue thus leading to obesity and how, on the contrary, obesity could result in insulin resistance [76]. Accordingly, obesity showed a similar effect to DM in ALS risk. With some exception in American studies [77], results converged into the hypothesis that elevated pre-diagnostic and baseline BMI scores are protective against both onset and survival of ALS and that conversely lower BMI is detrimental in developing the disease [7, 78]. A recent study showed that patients’ survival was related to the mean monthly percentage of weight loss at diagnosis (*p* < 0.0001) but not to pre-morbid BMI or BMI at diagnosis [79]. However, one study considered both obesity and DM and showed that DM was an independent prognostic factor on ALS risk [11].

In the setting of insulin resistance, the increased flux of FFAs to the liver results in increased triglyceride synthesis. This could justify the frequent co-occurrence of DM with the overproduction of very-low-density lipoproteins (VLDL), hypertriglyceridemia, and the reduction of HDL cholesterol [76]. Dyslipidemia together with a low-level inflammatory state that seems to even precede the appearance of insulin resistance [80] leads to vascular damage, arterial hypertension, and ultimately to a higher risk of cardiovascular diseases (CVDs) [76].

According to the inverse association of DM and ALS risk, hypercholesterolemia has been found less frequently among ALS patients than controls [14] and has been reported to be inversely associated with ALS onset [7]. Moreover, hyperlipidemia seems to delay the age of ALS onset and prolong life expectancy [27]. Further pieces of evidence showed lower frequencies of arterial hypertension, cardiac arrhythmia, and myocardial infarction among ALS patients [19].

However, results are inconsistent across literature and other studies reported no altered lipid levels in ALS patients [81]. A previous study showed no significant influence of hypertension, cardiac arrhythmia, and coronary heart disease on ALS survival [19] while two recent studies showed that the comorbidity of hypertension and heart disease was associated with reduced survival in ALS [23, 24]. Finally, it has been suggested that BMI rather than hyperlipidemia could influence ALS prognosis [78, 82].

Despite such metabolic abnormalities that could be read as epiphenomena of insulin resistance, a recent MR study demonstrated that dyslipidemia could be causative itself [83].

As a final remark, it remains unclear whether all metabolic findings could be a compensation for hypermetabolism found in ALS patients [5]. Despite hyperglycemia, early weight loss, and dyslipidemia could be read as compensation effect of a high energy expenditure, in the absence of studies on presymptomatic patients, it remains unclear if hypermetabolism is a product or a cause of the neurodegenerative process [84].

In conclusion, DM and ALS seem to be inversely correlated as concerning ALS risk; conversely, the findings on the effect of DM on ALS prognosis are inconclusive. The exact causal relationship between DM and ALS remains unclear. However, such a relationship should be read as the piece of a larger metabolic dysfunction and could be related to the finding of hypermetabolism in ALS patients. Further genetics studies using techniques such as MR could be helpful in disentangling this complex metabolic, possibly across different ethnic backgrounds.

## Data Availability

Data sharing is not applicable to this article as no new data were created or analyzed in the study.
